# Beyond Static Snapshots: Assessing Critical Thinking Ability Through Iterative Argumentation Processes

**DOI:** 10.3390/jintelligence14080190

**Published:** 2026-08-19

**Authors:** Liming Jiang, Fang Luo, Xuetao Tian

**Affiliations:** 1 Faculty of Psychology, Beijing Normal University, Beijing 100875, China; jiangliming@microsoft.com (L.J.); luof@bnu.edu.cn (F.L.); 2 Microsoft Research Asia, Beijing 100080, China

**Keywords:** critical thinking, iterative argumentation, process-based assessment, psychometric validation

## Abstract

In an era of information explosion and overload, individuals face a constant influx of mixed-quality, contradictory data. Navigating this complex landscape requires critical thinking, which enables individuals to dynamically adjust their reasoning and refine judgments. Consequently, accurately measuring this dynamic ability is essential. However, existing assessments predominantly focus on single-round argumentation and fail to capture the iterative process, potentially yielding unrepresentative evaluations of real-world performance. To address this gap, this study develops the Iterative Argumentation Task (IAT), a novel assessment tool that presents participants with a sequence of interrelated passages simulating real-world information flow. By capturing the process of evaluation, position changes, and argument revisions, the IAT evaluates three ability dimensions: single-round argument analysis, inter-argument relationship analysis, and argument integration. We recruited 355 undergraduates to validate the IAT. Confirmatory factor analysis provided evidence for the proposed measurement structure. Furthermore, correlations with established critical thinking and reasoning tests demonstrated convergent and discriminant validity, respectively. Moreover, performance differences between the undergraduates and 33 debaters established known-groups validity. Finally, latent profile analysis identified four distinct profiles across the IAT dimensions. Together, these findings establish the IAT as a theoretically sound and practically effective tool for assessing critical thinking.

## 1. Introduction

In an era characterized by information overload and the proliferation of diverse content sources, individuals are constantly faced with an influx of mixed-quality information, making it increasingly difficult to maintain rationality (Floridi, 2014; Shao et al., 2018). The rapid advancement of artificial intelligence has further intensified this complexity. Critical thinking, defined as a reflective way of thinking grounded in reliable evidence and rigorous reasoning (Ennis, 1993; Paul & Elder, 2014), enables individuals to discern credible information from misinformation and make sound judgments (Halpern, 2003). Given its pivotal role in navigating the complexities of the modern world, critical thinking has been identified as a core competency for the 21st century (Voogt & Roblin, 2012), especially in the age of artificial intelligence (Miao & Shiohira, 2024). Therefore, the development of a valid critical thinking assessment is imperative for evaluating this proficiency and ultimately fostering rational individuals.

Argumentation, the process of constructing claims, organizing evidence, and reasoning toward justified conclusions (Kuhn, 1991; Toulmin, 1958), is widely recognized as the primary vehicle through which critical thinking manifests and can be observed (Mercier & Sperber, 2011). It is through argumentation that individuals externalize their reasoning, making the quality of their analysis, evaluation, and inference accessible to assessment (Chen & She, 2012; Paul & Elder, 2014). Accordingly, most existing critical thinking instruments adopt argumentation-based formats (Liu et al., 2014).

However, existing critical thinking ability assessments predominantly focus on single-round argumentation, in which individuals analyze a limited set of information and draw conclusions in a single attempt. In contrast, real-world information emerges continuously and often contains contradictions (Dwyer, 2023), requiring individuals to dynamically update their reasoning. For instance, individuals may reassess their position on a social event as new details emerge or revise a purchasing decision upon encountering product reviews. Crucially, the ability to evaluate how new information relates to and challenges existing arguments and to engage in ongoing reflection to refine conclusions represents a core aspect of critical thinking (Dwyer et al., 2014; P. Facione, 1990; Phan, 2010). Yet current assessments, failing to capture these iterative processes, primarily identify individuals who perform well in static contexts, thereby yielding unrepresentative evaluations for real-world critical thinking performance.

This study addresses this gap by developing an Iterative Argumentation Task (IAT)^1^ that not only captures the critical thinking ability dimensions assessed by existing tests, but also encompasses the competencies demanded by iterative argumentation processes. The IAT presents participants with a sequence of pre-designed interrelated passages on a given topic to mirror the real-world information flow. Participants’ analysis and evaluation of passages, reasons for position changes, and processes of argument revision are all captured, from which fine-grained indicators are extracted to estimate their critical thinking ability. We systematically examined the psychometric properties of the IAT through multiple methods of reliability and validity assessment. Ultimately, this research seeks to shift the evaluation of critical thinking from static snapshots to process-based measurements, thereby providing a more authentic reflection of individuals’ performance in complex information environments.

## 2. Background

### 2.1. Critical Thinking and Iterative Argumentation

Critical thinking encompasses the ability to systematically analyze information, evaluate the credibility and relevance of evidence, construct reasoned arguments, and engage in reflective judgment (Ennis, 1987; P. Facione, 1990). These cognitive operations find their most direct expression in the process of argumentation (Kuhn, 1991; Mercier & Sperber, 2011). Indeed, argumentation serves as both a manifestation and a mechanism of critical thinking: it externalizes reasoning processes, making them subject to scrutiny and refinement (Deane, 2011).

Argumentation fundamentally involves two processes: argument evaluation and argument construction (Walton, 1998). These two processes capture the core operations of critical thinking. When individuals critically evaluate an argument (e.g., deciding if a report is biased), they must assess whether the claims are well supported, whether the reasoning is logically sound, and whether alternative explanations have been adequately considered. Conversely, when individuals construct their own arguments (e.g., explaining why one deserve a raise), they must identify relevant evidence, organize it into a coherent line of reasoning, and anticipate potential objections (Ennis, 1987). Kuhn (2018) characterized these as the “input” and “output” phases of critical thinking, noting that proficient critical thinkers must excel at both.

A crucial distinction must be drawn between single-round argumentation and iterative argumentation, which is essential for how critical thinking is operationalized and assessed. Single-round argumentation, in which individuals analyze a limited and static set of information to formulate a conclusion in one attempt, captures important processes of critical thinking such as evidence evaluation. Yet real-world argumentation is inherently iterative and dynamic, characterized by the continuous emergence of new information that may support or contradict existing positions (Dwyer, 2023; Kuhn & Moore, 2015), demanding constant integration of new and existing information (Paul & Elder, 2014). Iterative argumentation is not simply the repetition of multiple single-round argumentation; rather, it involves qualitatively distinct cognitive operations that are central to critical thinking such as integrating diverse evidence and identifying similarities, differences, and relationships among various arguments (Ennis, 1987; Kuhn, 1991). These behavioral characteristics transcend single-round argumentation and can only be effectively exhibited and assessed through iterative argumentation processes.

### 2.2. Existing Critical Thinking Ability Assessments

A key requirement for eliciting, capturing, and assessing critical thinking ability in iterative argumentation is the provision of multiple interrelated information sources rather than isolated, single-source materials. Most existing instruments, however, focus predominantly on single-round argumentation and fail to adequately address iterative argumentation processes.

Multiple-choice items are the most widely used format for critical thinking ability assessment (Liu et al., 2014). They typically target argumentation elements (e.g., identifying the main conclusion) or the relationships among those elements (e.g., detecting reasoning errors) within a single passage. For instance, in the critical thinking part of the Thinking Skill Assessment (TSA, Black et al., 2008), participants respond to each item independently. By design, multiple-choice tests cannot reflect the iterative argumentation process.

Open-ended items represent another commonly used format for critical thinking assessment (Liu et al., 2014) and fall into two broad categories. The first requires participants to evaluate others’ arguments. For instance, the Ennis–Weir Critical Thinking Essay Test (EWCTET, Ennis & Weir, 1985) asks participants to provide a well-reasoned critique of each given passage. Participants analyze each paragraph independently, with no need to consider connections across paragraphs; consequently, iterative argumentation is barely involved. The second category requires participants to construct their own arguments by developing and articulating a position on a given topic. Depending on whether source materials are provided, these tasks can be further divided into two types.

No-material tasks present only a topic or brief background information, and participants draw on personal knowledge and experience to construct their arguments, as in the analytical writing task of the Collegiate Learning Assessment (Klein et al., 2007). In such cases, responses rely heavily on the retrieval of prior knowledge and personal experience. Without the input of new information, there is little to prompt reflection or adjustment, and iterative argumentation is largely absent.

Multi-material tasks, such as those used in the College Level Examination Program (College of the Mainland, 2015), require participants to form a position based on multiple documents and use the information therein as evidence to construct an argument. Because the materials are interrelated, participants must engage in iterative reasoning: when encountering information that contradicts an earlier source, they need to reflect on the credibility of each source and potentially revise their prior judgments. However, current multi-material tasks present all materials at once and evaluate only the final submitted essay. This approach fails to capture participants’ thinking processes and corresponding ability indicators during iterative argumentation.

### 2.3. A Framework for Critical Thinking Ability in Iterative Argumentation

**Figure 1:**
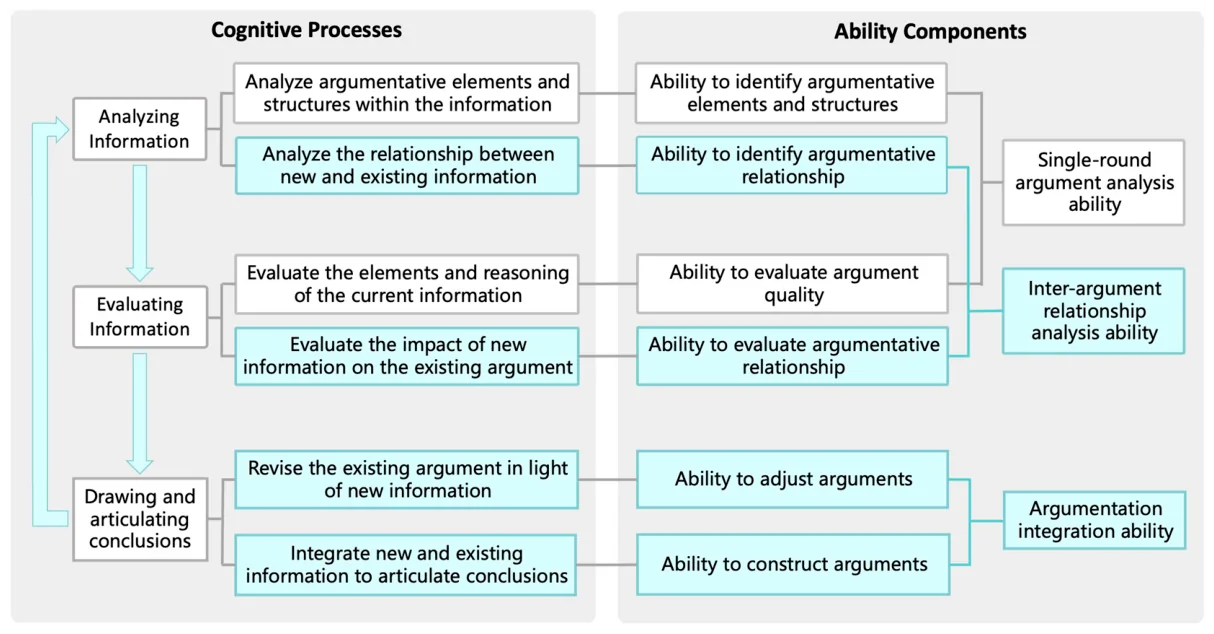
Cognitive processes and ability components in iterative argumentation comparing with single-round argumentation. Note: Blue boxes represent the elements unique to iterative argumentation (four key cognitive processes, four sub-abilities, and two higher-level abilities), whereas white boxes represent elements of single-round argumentation. The blue arrows depict the iterative progression across cognitive processes.

To address the limitations of existing assessments and to operationalize iterative argumentation for measurement, we synthesized a theoretical framework including cognitive processes and corresponding ability components. The critical thinking process in argumentation can be conceptualized into three core phases (as illustrated in Figure 1): analyzing information, evaluating information, and drawing and articulating conclusions. Among these, analyzing information and evaluating information constitute the input phase of critical thinking, whereas drawing and articulating conclusions represents the output phase (Kuhn, 2018).

- Analyzing information involves comprehending and deconstructing the content from an argumentative perspective. Within this phase, single-round argumentation is primarily reflected in the process of analyzing argumentative elements and structures, which captures the ability to identify argumentative elements (P. Facione, 1990). When extended to iterative argumentation, an additional process emerges: analyzing the relationships between new and existing information, which requires the ability to identify argumentative relationships (P. A. Facione & Gittens, 2015; Paul & Elder, 2014).
- Evaluating information focuses on assessing the strength of the argument. Single-round argumentation is primarily reflected in evaluating the elements and reasoning of the current argument, corresponding to the ability to evaluate argument quality (P. Facione, 1990). Iterative argumentation, however, extends this by introducing an additional process: evaluating the impact of new information on the existing argument, which corresponds to the ability to evaluate argumentative relationships (Bailin et al., 1999; King & Kitchener, 2004).
- Drawing and articulating conclusions entails forming a conclusion based on the information and defending it in an argumentative format. Within this phase, single-round argumentation mainly involves forming and articulating a position based on a single piece of evidence. In iterative argumentation, however, this phase becomes more complex and can be separated into two processes. The first process is revising the existing argument in response to new information. This process goes beyond mere addition; it requires incorporating new information into the existing argument through deliberate and appropriate integration (Bailin et al., 1999; King & Kitchener, 2004). For example, if new evidence contradicts a previously held piece of evidence, one must decide whether to discard the original evidence. Conversely, if new evidence supplements existing evidence, one may need to incorporate it into the argument. The second process is constructing an integrated argument that synthesizes all available information. Rather than focusing on the impact of individual pieces of new evidence, this process involves weaving together multiple sources into a coherent, logically consistent argument (P. A. Facione & Gittens, 2015; King & Kitchener, 2004; Voss & Means, 1991). For example, as evidence accumulates over multiple rounds, individuals must periodically step back to systematically re-examine all materials and determine whether their overall argument should be maintained, refined, or fundamentally revised.

## 3. Design

### 3.1. Assessment Task Overview

**Figure 2:**
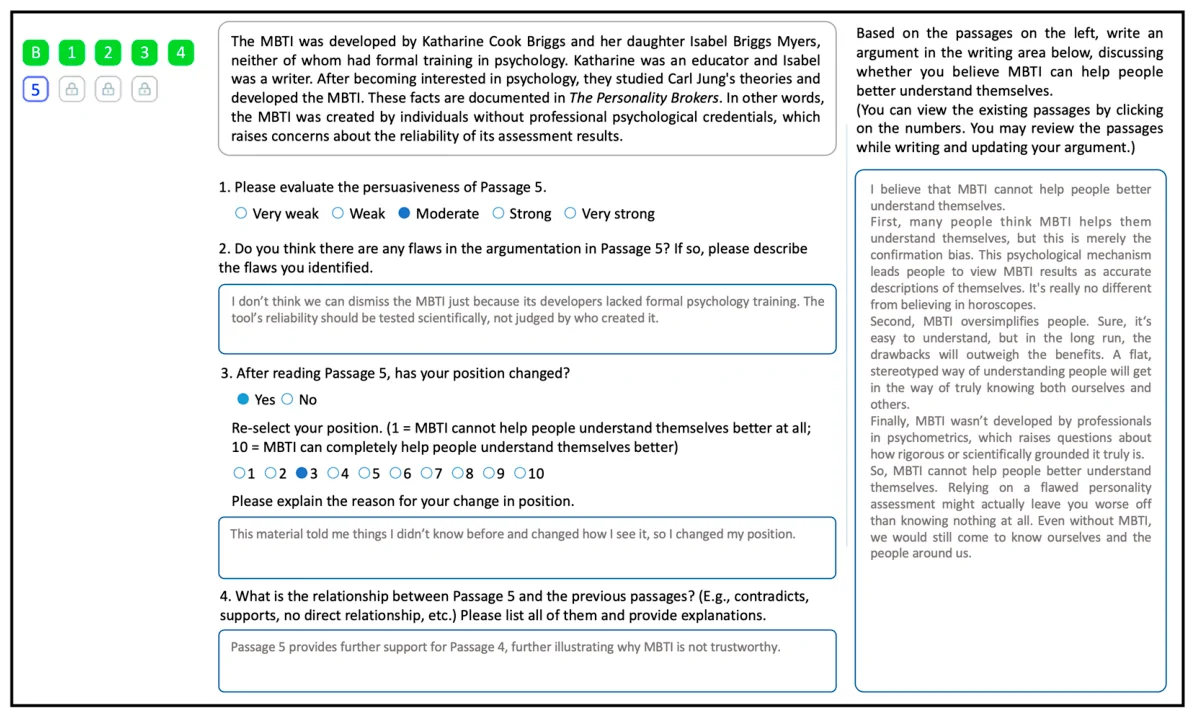
Interface of the Iterative Argumentation Task. Note: In the left panel, the “B” button stands for Background, which provides a brief introduction to the topic. The numbered buttons correspond to specific passages (e.g., “1” represents Passage 1). Green buttons indicate unlocked passages, whereas gray ones represent locked passages. The current passage is highlighted with a blue outline (i.e., Passage 5). Original interface in Chinese; translated into English here.

The iterative argumentation task (IAT) adopts a multi-round design in which passages on a specific topic are presented sequentially. In the present study, we utilized the topic “Can MBTI help people better understand themselves?” as illustrated in Figure 2. Participants begin by reading background information and indicating their initial position on the topic on a 10-point scale ranging from 1 (MBTI cannot help people understand themselves better at all) to 10 (MBTI can completely help people understand themselves better). They then engage with eight passages in sequence and complete items associated with each passage. After participants submit their responses, the next passage is unlocked. Throughout the task, participants may revisit earlier passages at any time, though previously submitted responses cannot be revised. In addition to the round-specific items, the IAT incorporates an argument writing item that runs throughout the assessment, asking participants to continuously refine their argument as new passages become available.

This design offers several core advantages. First, it simulates the incremental way in which people acquire and process information in everyday contexts, thereby enhancing the ecological validity of the assessment. Second, the response types are designed to elicit and record participants’ thinking in real time such as prompting them to articulate how the current passage relates to prior ones. Furthermore, the IAT captures process data that traditional measures rarely obtain, such as the revisions made to participants’ written arguments. Together, these diverse process data yield fine-grained indicators of critical thinking performance.

The topic “Can MBTI help people better understand themselves?” was selected as a representative issue for this assessment for several reasons. (1) It is an open-ended social issue with no definitively correct answer, which ensures freedom of expression. (2) The richness and diversity of materials on this topic provided a sufficient basis for constructing balanced argumentative passages representing both sides. (3) The topic is grounded in daily life and familiar among Chinese undergraduates, helping to minimize inter-individual differences in prior knowledge and ensure a comparable starting baseline for all participants. (4) The available materials encompass multiple viewpoints, providing a broad content base for the construction of argumentative relationships across passages.

Passages were balanced in number across both sides and were presented in alternating order to minimize order effects (e.g., primacy and recency effects; Miller & Campbell, 1959). This arrangement also serves to maximize cognitive conflict, thereby encouraging self-reflection and adjustment.

### 3.2. Response Types and Data Collected

**Figure 3:**
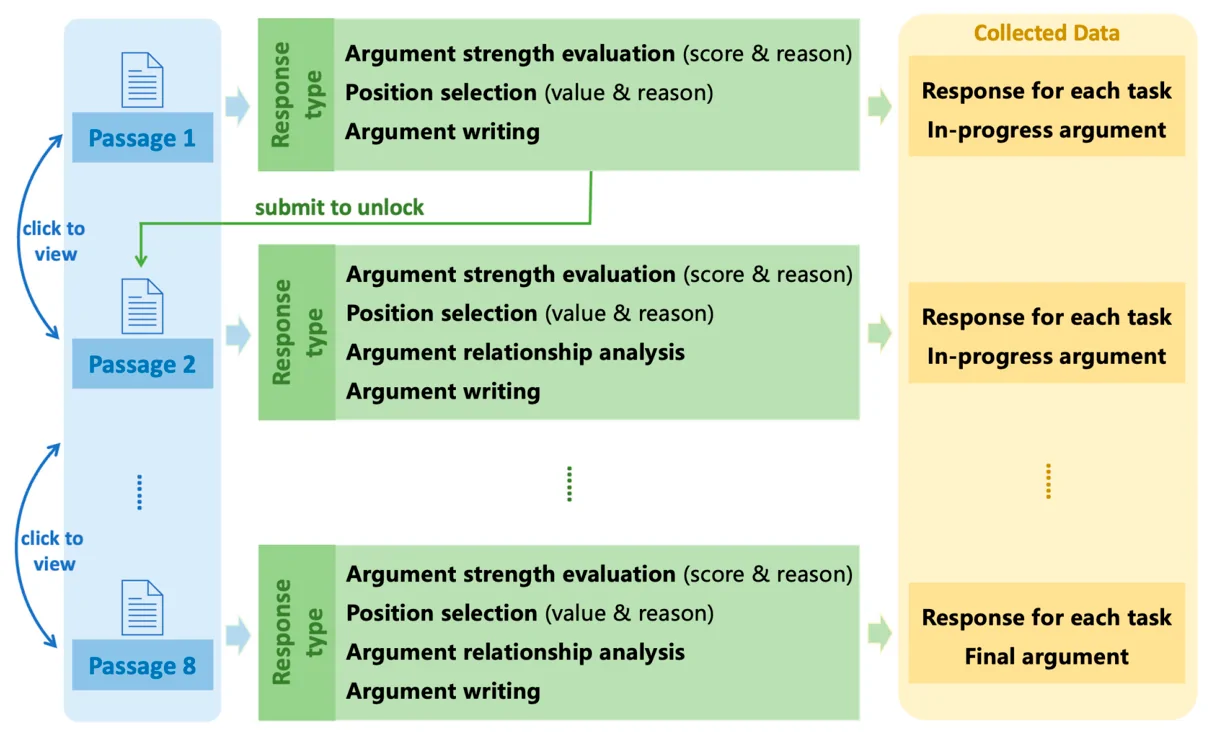
Overview of the IAT procedure, detailing the response types and collected data. Note: Blue boxes represent passages, green boxes indicate response types, and yellow boxes show the collected data.

The IAT comprises four response types: (1) argument strength evaluation, (2) argument relationship analysis, (3) position selection, and (4) argument writing. The first three types are associated with each passage (with the exception that passage 1 does not include the argument relationship analysis). The pipeline, response types, and collected data are presented in Figure 3.

(1) Argument strength evaluation: Participants evaluate the strength of each passage’s argument and provide reasons. Six passages (passages 1, 2, 3, 4, 5, and 8) contain predetermined argumentative flaws, such as overgeneralization or false analogy.

(2) Argument relationship analysis: Participants analyze the relationship between the current passage and previous passages. Five passages (passages 2, 4, 6, 7, and 8) contain predetermined relationships to other passages, such as contradicting the evidence in an earlier one.

(3) Position selection: In each round, participants determine whether their position on the topic has changed; if so, they select a new position and provide a reason for the change. If not, they provide a reason for maintaining it.

(4) Argument writing: Participants revise their written argument continuously and submit the final version at the end of the assessment. Each time a participant leaves the writing area, the system automatically saves the current text.

## 4. Methods

### 4.1. External Measures

Two established critical thinking ability assessments were administered as criterion measures: TSA (Black et al., 2008) and EWCTET (Ennis & Weir, 1985).

- The TSA, developed by the University of Cambridge as part of its admissions assessment, is an annually administered multiple-choice critical thinking test. Each item in the TSA presents participants with a short passage, a question, and five options, requiring them to read the text and select the most appropriate option to answer the question. Item types in the TSA include tasks such as identifying the assumption and applying the principle. For the current study, 18 items free of cultural bias were selected from the TSA item bank.
- The EWCTET presents participants with an eight-paragraph letter advocating for a city-wide ban on nighttime roadside parking. Each paragraph contains a built-in logical flaw, such as false analogies. Participants are required to evaluate each paragraph by identifying and articulating the flaws.

To examine discriminant validity, a reasoning test comprising number series reasoning (4 items), matrix reasoning (3 items), and spatial reasoning (3 items) selected from the International Cognitive Ability Resource (Condon & Revelle, 2014) was also administered, as critical thinking is regarded as encompassing more than mere reasoning (Halpern, 1998). Examples of criterion test items are in Appendix A. All original tests were translated into Chinese.

### 4.2. Participants and Procedure

A total of 383 first-year undergraduates (prior to declaring their majors) from a top-tier university in China were recruited. Participants completed the IAT (90-min time limit), the TSA (30-min time limit), the EWCTET (30-min time limit), and the reasoning test (20-min time limit) in order.

Response quality was assessed mainly through the open-ended items. Six participants whose final arguments on the IAT contained fewer than 150 words and exhibited incomplete content were excluded. Final arguments between 150 and 250 words were reviewed and showed no evident incompleteness. Although some responses longer than 250 words ended mid-sentence (likely due to the expiration of time), these were retained given their substantial length. This screening yielded 377 valid cases.

Among these 377 students, 22 had failed to complete at least one passage in the IAT (i.e., they did not unlock the passage). These participants were excluded, resulting in a final sample of 355 participants. These 355 participants had no missing or invalid responses on the TSA, EWCTET, or reasoning test.

Additionally, to examine known-groups validity, 33 s- and third-year undergraduates from the same university who had participated in debate competitions were recruited from diverse majors (e.g., literature, medicine, and mathematics) as a comparison group relative to the general sample. Both debate experience and higher grade level may provide opportunities to practice iterative argumentation in authentic contexts, rendering the debater sample a theoretically higher-performing comparison group. The debater sample completed the IAT, the TSA, and the EWCTET.

For all open-ended measures, including the IAT and the EWCTET, responses from both the general sample and the debater sample were scored by the same trained raters using identical scoring rubrics, with raters blind to group membership.

### 4.3. Scoring

#### 4.3.1. Scoring of IAT Indicators

**Figure 4:**
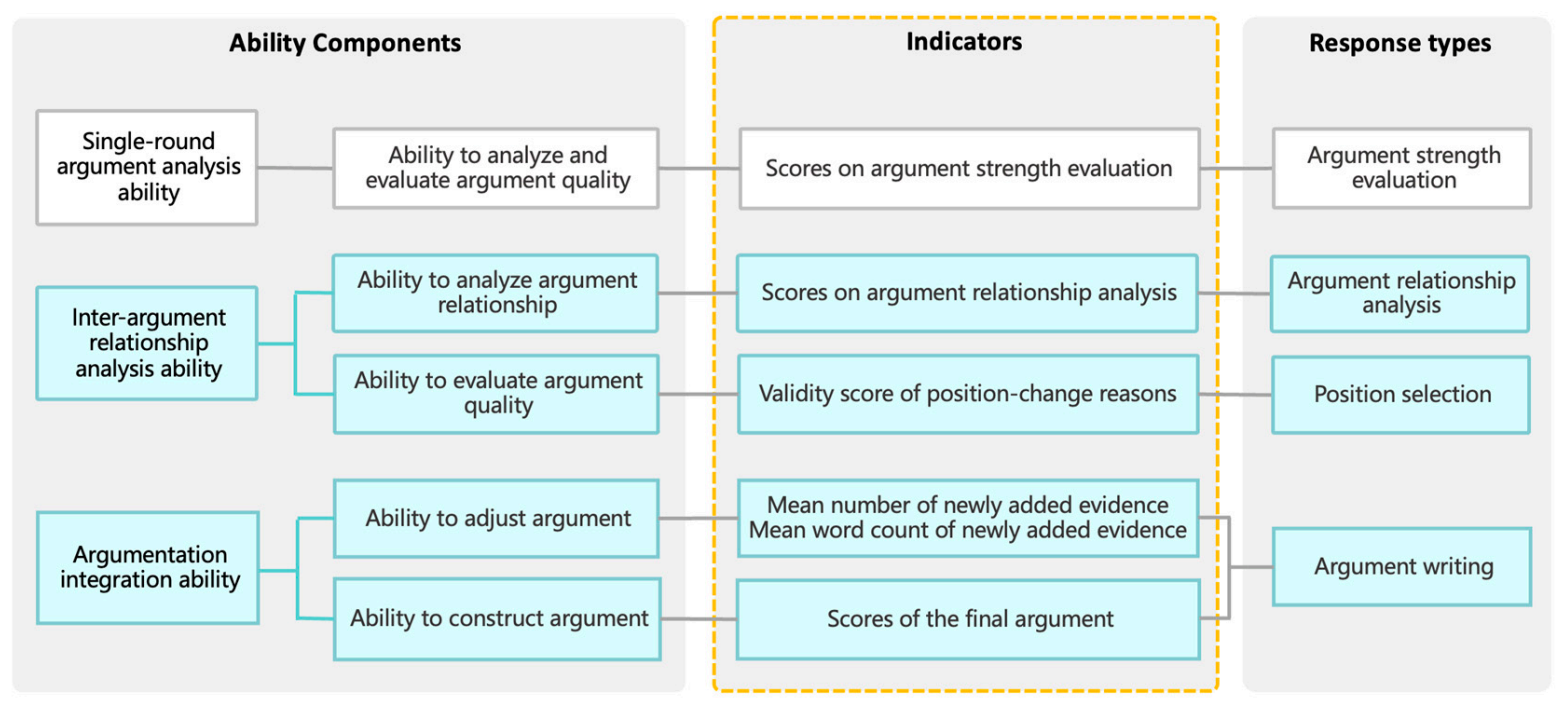
The correspondence between the ability components, indicators, and the response types. Note: “Ability to analyze and evaluate argument quality” in this figure is merged from “Ability to identify argumentative elements” and “Ability to evaluate argument quality” in Figure 1. The dashed orange box highlights the indicators used in this study.

Five categories of indicators were derived from the IAT, as presented in Figure 4. The following describes how each indicator was scored.

(1) Scores on argument strength evaluation: Participants’ responses were scored independently by two trained raters according to a rubric on a 0–3 scale per flaw. A score of 3 indicates that the response aligns with the key; scores of 2 or 1 indicate that the response addresses the correct aspect but lacks comprehensiveness or clarity; a score of 0 indicates that the response contradicts the key or is entirely irrelevant. Inter-rater reliability, assessed using intraclass correlation coefficients (ICC), ranged from 0.80 to 0.95 across passages, indicating acceptable consistency, with the mean score used as the final score.

(2) Scores on argument relationship analysis: Participants’ responses were scored independently by two trained raters according to a rubric on a 0–2 scale. One point is awarded for correctly identifying the relationship, with an additional point awarded for providing a correct reason. Inter-rater reliability, assessed using ICC, ranged from 0.82 to 0.94 across passages, indicating acceptable consistency, with the mean score used as the final score.

(3) Validity scores of position-change reasons: While the position change is subjective, the reasons provided for changing or maintaining one’s position vary in quality. Nine categories were developed using the constant comparative method (Glaser, 1965), whereby coding scheme refinement proceeded iteratively until theoretical saturation was reached (i.e., no new reason prompted further revision of the coding scheme). Final coding scheme and corresponding validity scores are shown in Table 1.

- Score of 0: The “consistency” category reflects a heuristic way of thinking in which participants judge whether to change their position based solely on whether the passage aligns with their pre-existing position. The “indirect rebuttal” category reflects an ignoring of the passage content, instead attempting to counter the argument by raising other irrelevant considerations. Both categories lack substantive engagement with the passage content and are therefore assigned a score of 0.
- Score of 1: The “prior knowledge” and the “new information” categories indicate that participants connected the passage content to existing knowledge or information, reflecting a relatively shallow level of content engagement. These categories are assigned a score of 1.
- Score of 2: The “endorsement” and the “direct rebuttal” categories reflect participants’ use of passage content to articulate why they agree or disagree with the argument, serving as the basis for their position decision. These categories represent the most in-depth engagement with the passage content and are therefore assigned a score of 2.
- Score of −1: Responses that provide no reason are coded as “invalid” and assigned a score of −1.

Three raters independently coded all reasons based on the finalized scheme. Cohen’s Kappa coefficients ranged from 0.72 to 0.91 across categories, indicating high inter-rater agreement. Final codes were determined by majority vote: a category was applied if at least two of the three coders endorsed it.

Because the reason provided in a single round can be subject to randomness (e.g., a participant may change their position without adequate justification simply because they feel uncomfortable with the passage content), validity scores from each passage were summed to yield an overall validity score.

(4) Mean number and mean word count of newly added evidence: By comparing in-progress versions of written arguments, we identified the content added in each revision. The eight passages collectively contained 11 evidence units. We used ERNIE-4, a large language model (LLM) popular in the Chinese context (Zhang et al., 2019), to code newly added evidence via API. Detailed coding procedures of the LLM are reported in Appendix B. Based on this coding, we calculated the mean number and the mean word count of evidence newly added per revision.

(5) Scores of the final argument: The final argument was evaluated across five dimensions:

- Clarity of claim: The argument presents a clear and explicit position.
- Sufficiency of evidence: The argument provides adequate and valid evidence to support the claim.
- Adequacy of elaboration: The argument sufficiently explains how the evidence supports the claim.
- Coherence of structure: The argument demonstrates a clear and logical structure.
- Fairness: The argument objectively considers both sides of the issue.

These dimensions were derived from a review of existing scoring rubrics for writing-based critical thinking assessments such as the Collegiate Learning Assessment (Aloisi & Callaghan, 2018) and the ETS Proficiency Profile (Liu et al., 2012). Three raters independently scored all the arguments, with each dimension rated on a 1–5 scale. The rubric is detailed in Appendix C. Inter-rater reliability was assessed using ICC; values ranged from 0.70 to 0.84 across five dimensions, indicating good inter-rater consistency. The mean score across raters was used as the final score for each dimension.

#### 4.3.2. Scoring of External Measures

The TSA and reasoning test both consisted of items with single correct answers and were scored accordingly. For the EWCTET, which is an open-ended measure, two trained raters independently scored all responses from both the general sample and the debater sample using the same scoring rubric, with the mean score used as the final score for each item. Inter-rater reliability, assessed using ICC, ranged from 0.85 to 0.95 across items, indicating good consistency.

### 4.4. Data Analysis

For the IAT, confirmatory Factor Analysis (CFA) was conducted to construct the measurement model, during which items exhibiting poor quality were removed. Structural validity was subsequently examined. Based on the final measurement model, internal-consistency reliability and internal convergent validity were assessed. Ability estimates derived from the final model were used as the basis for latent profile analysis (LPA), which was conducted to identify distinct ability profiles manifested in the IAT. Using the same ability estimates, correlational analyses were performed with scores from the TSA, EWCTET, and reasoning test to examine external convergent and discriminant validity. To examine known-groups validity, ability levels between the general sample and the debater sample were compared by obtaining ability estimates for the debater sample within a measurement invariance framework, which placed latent scores from both groups on a common scale. Note that all preceding analyses were based on the general sample (*n* = 355); the debater sample was introduced solely for known-groups validity examination.

For the TSA and EWCTET, measurement models were constructed by CFA based on the general sample to obtain factor scores, which were then applied to the debater sample for group comparisons (see Appendix A for detailed information).

All statistical analyses were performed using R version 4.5.3. All data and code used in this study are openly available at: https://github.com/Jiang-Liming/Materials-for-IAT-paper (accessed on 16 August 2026).

## 5. Results

### 5.1. Description of IAT Indicators

Table 2 presents the description of each indicator in the IAT based on the MBTI topic. The pass rates for the argument strength evaluation ranged from 0.11 to 0.79, with an average pass rate of 0.42, while the pass rates for the argument relationship analysis ranged from 0.18 to 0.52, with an average pass rate of 0.42. For these two types of response with correct answers, the difficulty levels were well-distributed and moderate.

Among the five scoring dimensions of the argument writing, the scores for clarity of claim (M = 4.26) and coherence of structure (M = 3.47) skewed toward the higher end. These two outcomes are likely attributable to argument writing training in high school education, which teaches students that an argument should present a clear claim and structure. A common structure includes the first paragraph stating a claim, the second paragraph elaborating reasons supporting one’s claim, the third paragraph refuting opposing viewpoints, and the final paragraph providing a conclusion. Mean scores for the remaining dimensions ranged between 2.5 and 3, indicating that the scoring rubrics were appropriately calibrated to capture meaningful variation in performance.

**Figure 5:**
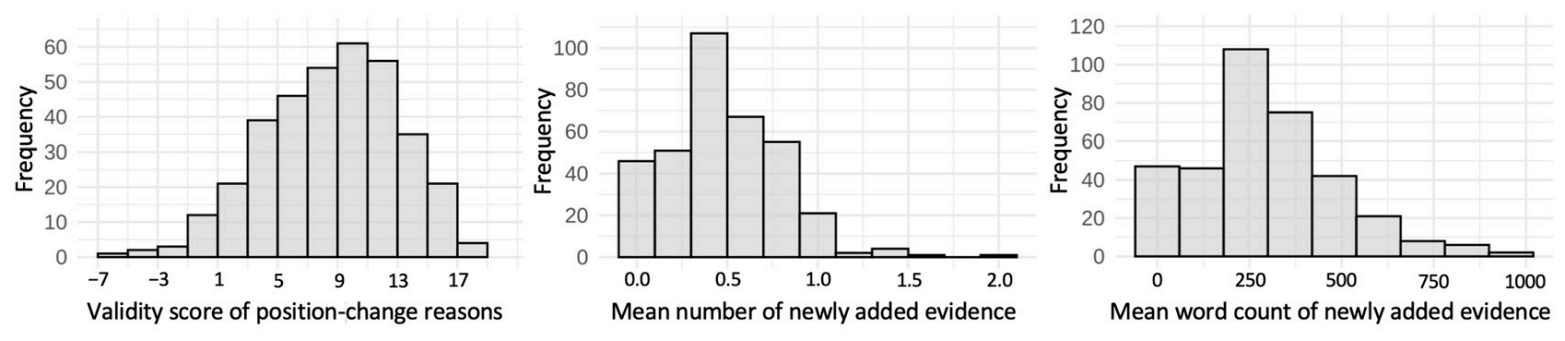
Distribution of three IAT indicators (validity score of position-change reasons, mean number, and mean word count of newly added evidence).

Figure 5 shows that the validity scores for position-change reasons are approximately normally distributed (M = 9.06). In contrast, the two evidence-use indices (mean count of newly added evidence and mean word count of newly added evidence) exhibit positive skew. This skew is driven by a small subset of participants who either added many evidence items at once or provided lengthy elaboration around specific pieces of evidence; manual checks confirmed that these extreme cases were valid responses. Notably, both indices also reveal a clustering of scores at zero, indicating that some participants used no evidence in their written arguments.

### 5.2. Psychometric Properties

#### 5.2.1. Structural Validity

**Figure 6:**
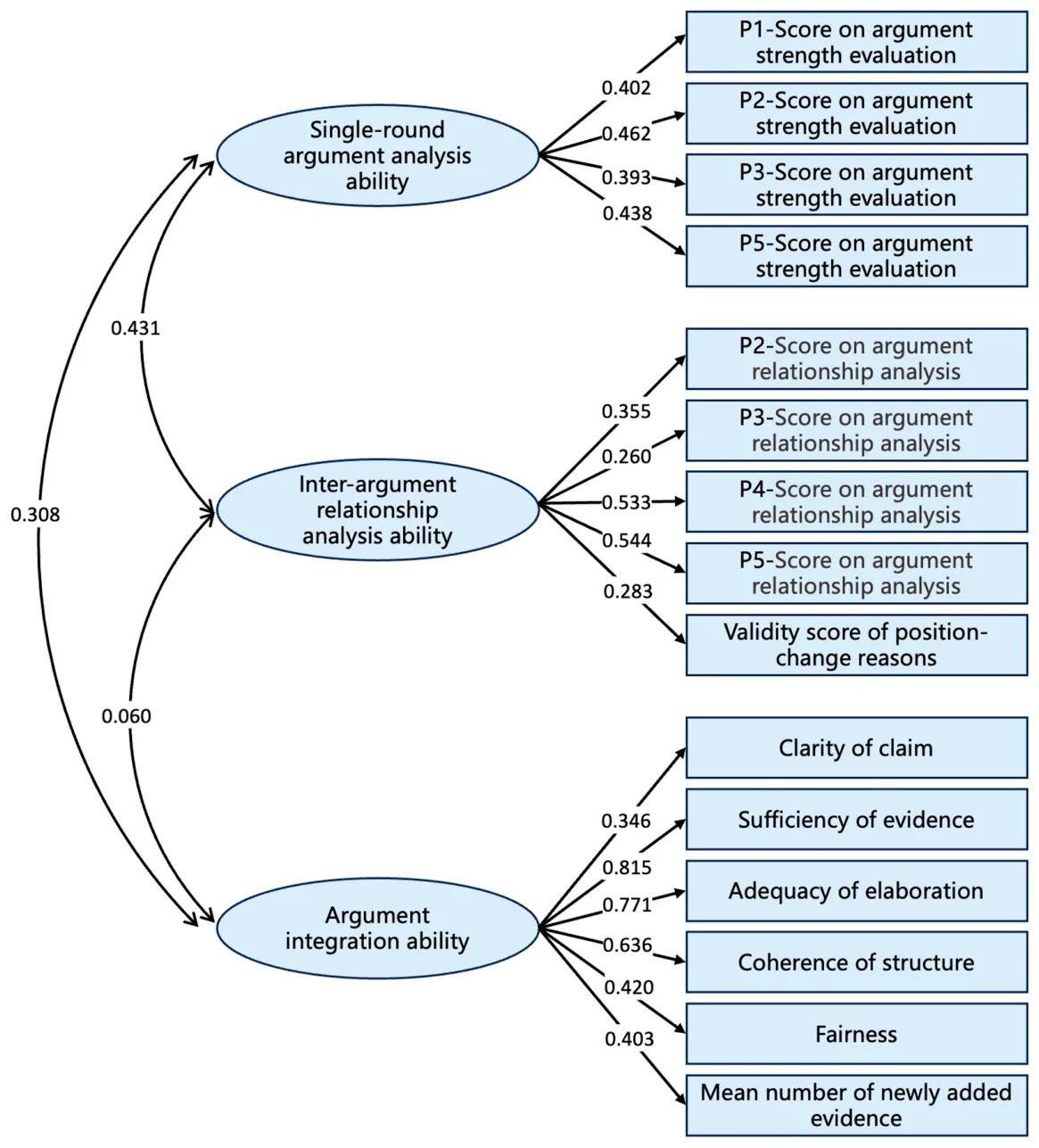
CFA model of IAT. Note: P1 to P5 means passage 1 to passage 5.

Confirmatory factor analysis (CFA) was employed to examine the relationships between the three ability dimensions and their corresponding indicators. In the initial model, three passage-specific indicators yielded low factor loadings: argument strength evaluation from Passage 4 (0.187), argument strength evaluation from Passage 8 (0.151), and argument relationship analysis from Passage 8 (0.197). Additionally, the variance of mean word count of newly added evidence was at least 1000 times larger than those of the other indicators, which may have introduced instability into the CFA estimation. Accordingly, these four indicators were excluded from the measurement model. The final model, presented in Figure 6, demonstrates good overall model fit (*χ*^2^ = 126.275, *df* = 87, *p* = .004, CFI = 0.937, TLI = 0.924, RMSEA = 0.036, SRMR = 0.048). These findings provide evidence for the structural validity of the hypothesized measurement model within the context of the current topic.

Based on this model, factor scores of the three ability dimensions were estimated. The correlation between single-round argument analysis ability and inter-argument relationship analysis ability was moderate (r = 0.43, *p* < .001). Both argument strength evaluation and argument relationship analysis are based on the comprehension and analysis of the same content, which likely accounts for the moderate correlation between the two. Single-round argument analysis ability showed a weaker, yet significant correlation with argument integration ability (r = 0.31, *p* < .001). Accurate evaluation of passages can, to some extent, inform the quality of argument writing; however, the two ability dimensions differ in their primary objectives, as the former emphasizes critically analyzing others’ arguments while the latter emphasizes constructing one’s own, which may account for the relatively weak association. Inter-argument relationship analysis ability and argument integration ability were nearly uncorrelated (r = 0.06, *p* = .260), suggesting that the two involve fundamentally distinct cognitive processes and ability components.

**Figure 7:**
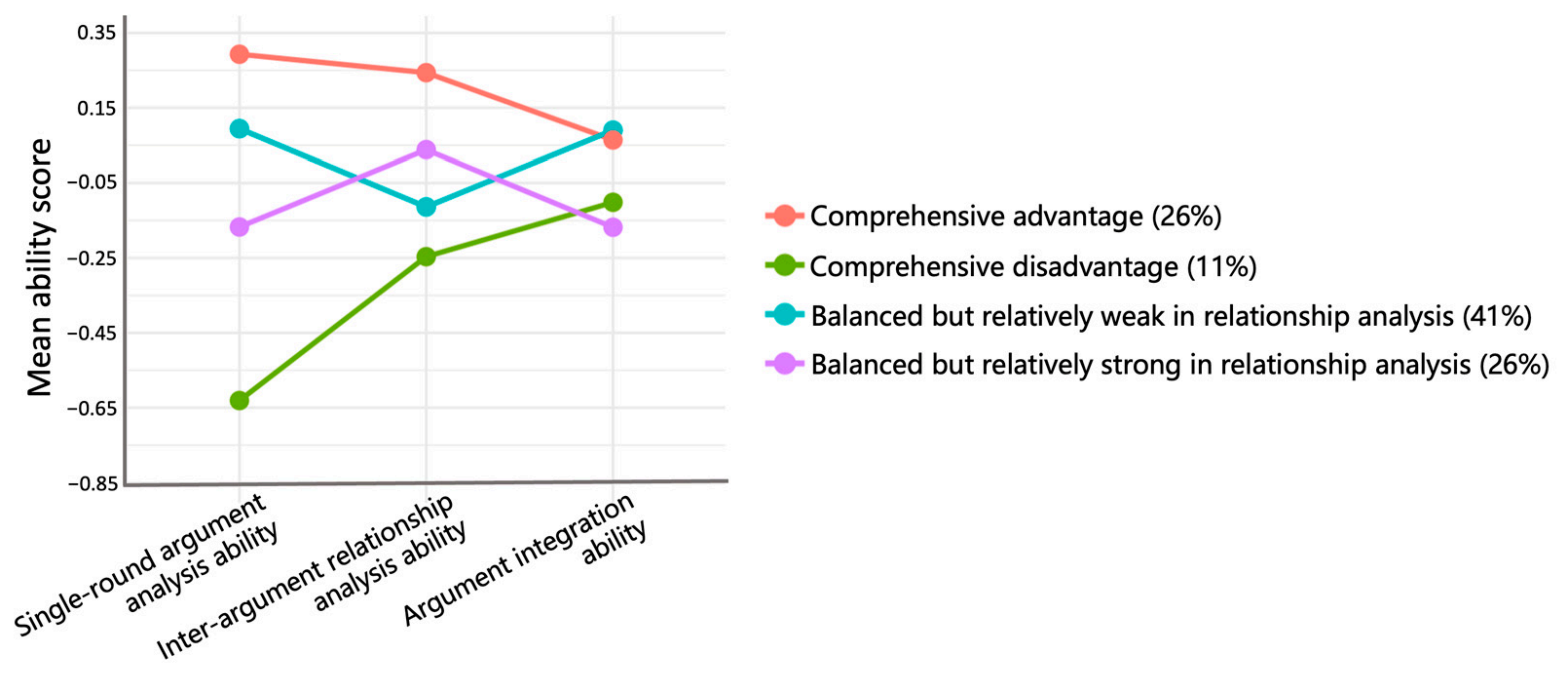
Mean abilities of four profiles from LPA.

Latent profile analysis (LPA) was conducted based on participants’ scores on three ability dimensions estimated by CFA to investigate the distinct critical thinking ability profiles emerging in iterative argumentation. Based on multiple model fit indices such as AIC, BIC, and Entropy (Tein et al., 2013), the four-profile model was identified as the optimal solution. The mean abilities across the four profiles are shown in Figure 7.

Profile 1: Comprehensive advantage (26% of general sample). This profile represents a broad advantage across all ability dimensions. This profile is characterized by the highest ability level on single-round argument analysis and inter-argument relationship analysis across all profiles, and a relatively high level on argument integration.

Profile 2: Comprehensive disadvantage (11% of general sample). Opposite to Profile 1, this profile exhibits broad deficits across all dimensions. This group presents the lowest ability level on single-round argument analysis and inter-argument relationship analysis, and a comparatively weak level on argument integration.

Profile 3: Balanced but relatively weak in relationship analysis (41% of general sample). This is the largest profile group. Participants with this profile display relatively high single-round argument analysis ability (though below Profile 1), slightly lower inter-argument relationship analysis ability that nonetheless exceeds that of Profile 2, and high argument integration ability.

Profile 4: Balanced but relatively strong in relationship analysis (21% of general sample). This profile presents an opposite pattern to Profile 3. Participants show slightly lower single-round argument analysis ability, though still above Profile 2, and slightly higher inter-argument relationship analysis ability that does not reach the level of Profile 1, and low argument integration ability.

Taken together, the LPA results reveal meaningful heterogeneity in participants’ critical thinking profiles during iterative argumentation. Beyond overall performance differences captured by Profiles 1 and 2, the contrast between the two balanced profiles (Profiles 3 and 4) further highlights that participants differed not only in general ability level but also in the configuration of strengths and weaknesses across the three dimensions.

To further investigate the characteristics of the identified profiles, we compared the four groups on external measures. As shown in Table 3, Profile 1 (comprehensive advantage) exhibited the strongest performance across criterion measures, whereas Profile 2 (comprehensive disadvantage) demonstrated the weakest performance. The two balanced groups showed little differences on criterion measures.

The differences in criterion measure performance across four profiles were examined by one-way ANOVA. Significant results emerged for both the TSA (*F*(3,351) = 8.22, *p* < .001) and the EWCTET (*F*(3,351) = 7.92, *p* < .001). Tukey HSD post hoc comparisons revealed that, for the TSA, Profile 2 scored lower than Profile 3 (△M = 0.06, *p* < .001), Profile 1 (△M = 0.07, *p* < .001), and Profile 4 (△M = 0.05, *p* = .007). Similarly, for the EWCTET, Profile 2 underperformed relative to Profile 3 (△M = 0.07, *p* = .012) and Profile 1 (△M = 0.12, *p* < .001).

Regarding the reasoning test, significant effects were found for number series reasoning (*F*(3,351) = 6.52, *p* < .001) and matrix reasoning (*F*(3,351) = 6.66, *p* < .001). Post hoc tests indicated that Profile 2 consistently underperformed compared to other profiles. For number series reasoning, Profile 2 scored lower than Profile 3 (△M = 0.05, *p* = .005), Profile 1 (△M = 0.07, *p* < .001), and Profile 4 (△M = 0.07, *p* < .001). For matrix reasoning, Profile 2 scored lower than Profile 3 (△M = 0.08, *p* = .003), Profile 1 (△M = 0.10, *p* < .001), and Profile 4 (△M = 0.10, *p* < .001). However, no significant profile effect was observed for spatial reasoning (*F*(3,351) = 1.70, *p* = .167).

#### 5.2.2. Reliability and Internal Convergent Validity

Building upon the final measurement model, the internal-consistency reliability and internal convergent validity of the three ability dimensions were evaluated using composite reliability (CR) and average variance extracted (AVE), respectively. CR values for single-round argument analysis, inter-argument relationship analysis, and argument integration were 0.452, 0.249, and 0.751, with only argument integration exceeding the recommended threshold of 0.70. AVE values for the three dimensions were 0.179, 0.093, and 0.373, none of which reached the threshold of 0.50.

These relatively low values may reflect, at least in part, the challenges of applying conventional psychometric indices to ecologically oriented, performance-based assessments (Linn et al., 1991). The IAT was designed to capture students’ performance in real-world argumentative tasks, where task-specific variance may reduce the shared variance among indicators (Shavelson et al., 1993). The limited number of indicators within each dimension may have further constrained the magnitude and stability of these estimates. Together, these findings point to the need for further refinement and expansion of the indicator structure in future iterations of the instrument.

#### 5.2.3. External Convergent and Discriminant Validity

Convergent and discriminant validity were examined by computing correlations between the criterion measures (TSA, EWCTET, and reasoning test) and the ability dimensions estimated within this specific topic context. As presented in Table 4, single-round argument analysis ability showed a modest correlation with the EWCTET (r = 0.33, *p* < .001) and a similar level of correlation with the TSA (r = 0.28, *p* < .001). Although these coefficients are modest, they are comparable to the correlation between the EWCTET and TSA (r = 0.033, *p* < .001), providing evidence of acceptable convergent validity.

Inter-argument relationship analysis ability showed weaker correlations with the TSA (r = 0.23, *p* < .001) and EWCTET (r = 0.22, *p* < .001) than did single-round argument analysis ability, which is largely attributable to the fact that inter-argument relationship analysis represents a novel aspect not captured by these traditional measures. Notably, argument integration ability showed the lowest correlations with the TSA (r = 0.11, *p* = .038) and EWCTET (r = 0.12, *p* = .029) among the three ability dimensions. Although the EWCTET also employs an open-ended response format, it only requires participants to evaluate given passages, whereas the argument integration requires them to construct and defend their own position. Framed within Kuhn’s (2018) distinction, the TSA, EWCTET, single-round argument analysis, and inter-argument relationship analysis all primarily involve the input phase of critical thinking, whereas argument integration primarily reflects the output phase. This theoretical distinction explains the low correlations between argument integration ability and the other dimensions as well as the criterion measures.

Finally, all three ability dimensions in the IAT showed weak correlations with the reasoning test, ranging from −0.04 to 0.22. This pattern suggests that argument-based critical thinking performance is primarily a product of education and training (Kuhn, 1991; Brookfield, 2011) rather than a direct reflection of basic cognitive ability. These findings support the discriminant validity of the IAT.

#### 5.2.4. Known-Groups Validity

Prior to comparing the three IAT ability dimensions between the general and debater samples, measurement invariance was examined. Metric invariance was supported relative to the configural model, Δ*χ*^2^(12) = 17.75, *p* = .124. Full scalar invariance was not achieved; accordingly, two non-invariant intercepts (“argument strength evaluation for Passage 5” and “mean number of newly added evidence”) were allowed to vary across groups. The resulting partial scalar invariance model did not significantly worsen model fit relative to the metric model, Δ*χ*^2^(10) = 10.06, *p* = .435. Ability estimates used in the known-groups comparison were therefore derived from this partial scalar invariance model.

As shown in Table 5, participants with debate competition experience significantly outperformed the general sample on all three dimensions of the IAT under the current topic (Welch’s *t*-tests were used due to unequal variances): single-round argument analysis ability, *t*(37.97) = 8.19, *p* < .001, *d* = 1.51 (large effect size); inter-argument relationship analysis ability, *t*(33.95) = 5.78, *p* < .001, *d* = 1.66 (large effect size); and argument integration ability, *t*(36.86) = 3.67, *p* = .001, *d* = 0.74 (large effect size). They also demonstrated significantly higher performance on the EWCTET, *t*(46.63) = 29.33, *p* < .001, *d* = 3.83 (large effect size). In contrast, the two groups showed comparable performance on the TSA, *t*(40.60) = −1.40, *p* = .170, *d* = 0.22 (small effect size).

The TSA is designed to assess discrete cognitive skills required by critical thinking. The EWCTET and the IAT, by contrast, require participants to draw on and integrate multiple critical thinking skills within an open, realistic problem context. Debate training and advanced undergraduate education are both oriented toward the capacity to apply critical thinking in authentic, integrative situations rather than to improve isolated skills (Ku, 2009; Liu et al., 2014). The observed group difference therefore provides evidence for the known-groups validity of the IAT by showing its sensitivity to theoretically expected performance differences.

When the debater sample was incorporated into the LPA, the model yielded the same four profiles. Table 6 presents the proportion of the four profiles in the debater sample and the general sample. Notably, Profile 1 accounted for the largest proportion of the debater sample at 76%, compared with 26% in the general sample. Profile 2 accounted for 3% in the debater sample but accounted for 11% in the general sample. These results indicate that individuals with debate competition experience exhibit superior performance on the IAT, providing evidence for its known-groups validity.

Additionally, comparing the two balanced profiles within the debater sample reveals that a non-trivial proportion are relatively weak in inter-argument relationship analysis. This suggests that analyzing inter-argument relationships remains a challenging task, even for individuals with debate training.

## 6. Discussion

Critical thinking is inherently complex and multi-faceted, encompassing different cognitive processes and ability components (P. Facione, 1990; Halpern, 2003). Although various assessment instruments have been developed to tap into different processes and components (Liu et al., 2014), the absence of a unified framework has made it difficult to clarify how these components are related and how they jointly constitute critical thinking. The present study addresses this issue by proposing the iterative argumentation process as a framework. Within this framework, we systematically decompose the cognitive demands of both single-round argument and inter-argument processes, along with their corresponding ability components.

The relations among the three IAT dimensions provide evidence for this decomposition. Single-round argument analysis ability and inter-argument relationship analysis ability were moderately correlated. This association likely reflects their shared requirement for analyzing given arguments, but the two dimensions differ in analytical scope. Single-round argument analysis focuses on the quality and structure of an individual argument, whereas inter-argument relationship analysis requires participants to compare multiple arguments and identify relations among them.

In contrast, single-round argument analysis ability showed a weaker correlation with argument integration ability. This pattern is consistent with the distinction between evaluating others’ arguments and constructing one’s own argument. While the evaluation of source passages can inform argument writing quality, the two dimensions differ fundamentally in their objectives: the former emphasizes critically analyzing others’ arguments while the latter emphasizes constructing one’s own. This distinction aligns with prior research indicating that argument comprehension and production are only moderately correlated (Reznitskaya & Wilkinson, 2019), and that analytical critical thinking and argument writing show only a moderate association (Preiss et al., 2013). The present findings extend this distinction to the IAT context, suggesting that analytical and productive components of critical thinking are related but not interchangeable.

The near-zero correlation between inter-argument relationship analysis ability and argument integration ability further highlights the distinction between objectively mapping relations among arguments and strategically using arguments for defending one’s own stance. This divergence may stem from the fact that participants, engaged in an inherently goal-driven writing process (Hayes, 2012), tend to build upon conclusions. They dismantle source texts and reconfigure selected content to serve their own purposes (Spivey, 1990), focusing solely on how each extracted piece strengthens their claims.

Taken together, the observed pattern of correlations provides support for the theoretical perspective that critical thinking encompasses input and output phases, with each phase primarily recruiting distinct aspects of critical thinking ability (Kuhn, 2018). Because both single-round argument analysis ability and inter-argument relationship analysis ability fall within the input phase, they exhibit a moderate correlation. In contrast, argument integration ability operates within the output phase, and shows weaker associations with the former two dimensions.

Validity analyses of the IAT provided evidence that it captures aspects of critical thinking that extend beyond the scope of traditional, single-round argumentation instruments. First, correlations between the IAT ability dimensions and criterion measures revealed that single-round argument analysis ability showed the strongest associations with critical thinking criterion tests among the three dimensions, suggesting the overlap between this dimension and conventional instruments that focus on single-round argumentation. In contrast, inter-argument relationship analysis ability and argument integration ability, both involving the inter-argument analysis process, showed weaker correlations with these criterion measures. Second, LPA results indicated that a substantial proportion of participants demonstrated strong single-round argument analysis ability but weak inter-argument relationship analysis ability, further supporting the view that proficiency in single-round argumentation does not necessarily generalize to iterative argumentative contexts.

This distinction carries practical significance, as real-world critical thinking often demands navigating competing positions rather than evaluating arguments in isolation. The known-groups results provide support for this point: while the debater sample performed comparably to the general sample on the TSA, they significantly outperformed the general sample across all three IAT dimensions, with Profile 1 being substantially more prevalent among them. Together, these findings suggest that the IAT provides discriminative information beyond what traditional assessments offer, by capturing the comprehensive cognitive processes involved in authentic iterative argumentation.

Among the identified profiles, Profile 3 warrants particular attention given its prevalence and educational implications. The pattern of generally balanced performance alongside relatively weaker inter-argument relationship analysis ability may be partly explained by students’ prior learning experiences. Before entering university, students typically receive limited instruction on comparing, evaluating, and synthesizing competing arguments across multiple sources; instead, they are more likely to have practiced analyzing individual arguments and constructing evidence-based writing in support of certain position. While such experiences support the development of single-argument analysis skills, they provide less scaffolding for the relational processing required to coordinate competing arguments across sources. This pattern may further reflect the task-specific nature of argumentation self-efficacy (Allagui, 2024): confidence cultivated through familiar single-argument tasks does not readily transfer to less familiar multi-source argumentation contexts. These findings suggest that cross-argument coordination deserves more deliberate instructional attention, given evidence that critical thinking is amenable to targeted instruction and training (Abrami et al., 2008; Niu et al., 2013).

Finally, this study represents an advancement toward the process-based assessment of critical thinking. As a higher-order thinking ability, critical thinking necessitates process-oriented evaluation and research (Dwyer et al., 2014) because many crucial manifestations of critical thinking cannot be adequately captured through outcome measures alone. Currently, process-based research on critical thinking remains relatively limited, though it has been gradually advancing with the proliferation of various process data collection technologies (Cui et al., 2025). Effective process-based assessment and research necessitates technology for recording and analyzing process data. For instance, by embedding automated data collection mechanisms directly into the assessment platform, this study captured granular, in-process writing data. These data provide a crucial foundation for understanding participants’ iterative argumentation and revision processes. Moreover, to address the challenge of analyzing process data, which are inherently more voluminous and unstructured than traditional outcome metrics, this study leveraged the advanced text comprehension and instruction-following capabilities of the LLMs (Radford et al., 2019; Wei et al., 2022). By utilizing LLMs to identify specific evidence within the in-process writing texts, this approach achieved substantial gains in analytical efficiency without compromising accuracy.

## 7. Limitations and Future Directions

Several limitations of the present study warrant consideration and provide avenues for future research. First, due to practical limitations, such as administration time constraints and testing burden, the present study employed only a single topic for measurement. While the use of the MBTI topic provided a rich, ill-structured context for argumentation, employing a single topic limits the ability to disentangle general critical thinking skills from topic-specific effects. Future research should incorporate multiple, diverse topics to examine whether the measurement indicators, relations among IAT dimensions, and observed cognitive profiles remain stable across different contexts, thereby enhancing the generalizability of the findings.

Second, the current test development process is resource-intensive, requiring meticulous topic selection, material curation, and pilot testing. To mitigate item exposure and facilitate scalable application, future work needs to establish a standardized, and potentially automated, framework for test construction. This advancement would not only streamline development efficiency but also provide a crucial foundation for ensuring cross-topic equivalence.

Third, the relatively small debater sample limited statistical power and may have reduced the stability of the observed group differences and effect sizes; findings involving this sample should therefore be interpreted with caution and regarded as preliminary. In addition, the present study did not isolate the effects of debate experience from those of undergraduate education. Future research could disentangle these factors to clarify how different educational experiences shape performance across the three IAT dimensions.

Finally, because the IAT captures complex cognitive dimensions often missed by existing instruments, establishing its predictive validity against real-world criteria is paramount. Future research should incorporate more diverse ecological metrics such as long-term academic or professional performance to confirm whether the IAT offers superior predictive power for real-world critical thinking outcomes.

## 8. Conclusions

This study developed the Iterative Argumentation Task (IAT) to address the limitations of static assessments and measure dynamic critical thinking in complex information environments. By simulating real-world information flows with interrelated passages, the IAT effectively captures the iterative processes and measures corresponding ability dimensions of critical thinking. Our findings provide evidence for the psychometric properties of the IAT and identify distinct ability profiles, indicating its incremental value in capturing aspects that may be less visible in traditional static tests. Despite potential limitations such as relying on a single topic, this study provides valuable insights into the theoretical foundations and the practical application of critical thinking assessments.

## Figures and Tables

**Table 1:** Categories and examples of position-change reasons.

Score	Category	Explanation	Example
2	Endorsement	Endorsement of the passage content	“The person in this passage learned about their personality through MBTI and found greater success in both work and personal life. It convinced me that MBTI can help people better understand themselves.”
2	Direct rebuttal	Direct rebuttal of the passage content	“The way large companies use MBTI must differ from how individuals use it. It is a mistake to claim that its benefits outweigh its drawbacks. So I maintain my position.”
1	Prior knowledge	Passage content already considered	“I was already aware that some large companies use MBTI; this passage did not change my position.”
1	New information	New information introduced by the passage	“It broadened my understanding and thereby influenced my judgment, which is why my position shifted.”
1	High persuasiveness	High persuasiveness of the passage	“The passage offers a profound examination of human psychological factors and is thus highly persuasive.”
1	Low persuasiveness	Low persuasiveness of the passage	“The passage contains flaws and is therefore not credible. I will not change my position.”
0	Consistency	Passage consistent with prior position or view	“The passage largely aligns with my existing view, so my position remains unchanged.”
0	Indirect rebuttal	Rebuttal based on irrelevant factors rather than passage content	“Although MBTI has this limitation, it remains a powerful tool for people to understand themselves.”
−1	Invalid	No substantive reason provided	“I’ll keep my view.”

**Table 2:** Description of IAT Indicators.

Indicator	Mean	SD	Min	Max	Full Score	Mean Proportion of Maximum Score
P1-Score on argument strength evaluation	4.73	1.35	0.00	6.00	6.00	0.79
P2-Score on argument strength evaluation	2.01	1.09	0.00	3.00	3.00	0.67
P3-Score on argument strength evaluation	0.94	0.83	0.00	3.00	3.00	0.31
P4-Score on argument strength evaluation	0.42	0.59	0.00	2.50	3.00	0.14
P5-Score on argument strength evaluation	1.41	0.74	0.00	3.00	3.00	0.47
P8-Score on argument strength evaluation	0.34	0.76	0.00	3.00	3.00	0.11
P2-Score on argument relationship analysis	1.54	0.80	0.00	3.00	3.00	0.51
P4-Score on argument relationship analysis	0.88	0.69	0.00	2.00	2.00	0.44
P6-Score on argument relationship analysis	1.04	0.83	0.00	2.00	2.00	0.52
P7-Score on argument relationship analysis	0.89	0.75	0.00	2.00	2.00	0.45
P8-Score on argument relationship analysis	0.72	0.97	0.00	4.00	4.00	0.18
Validity score of position-change reasons	9.06	4.41	−6.00	19.00	-	-
Clarity of claim	4.26	0.75	1.00	5.00	5.00	0.85
Sufficiency of evidence	2.92	0.70	1.00	4.33	5.00	0.58
Adequacy of elaboration	2.72	0.74	1.00	4.67	5.00	0.54
Coherence of structure	3.47	0.67	1.33	5.00	5.00	0.69
Fairness	2.96	0.75	1.00	4.33	5.00	0.59
Mean number of newly added evidence	0.49	0.32	0.00	2.00	-	-
Mean word count of newly added evidence	293.44	192.71	0.00	989.00	-	-

Note: Mean proportion of maximum score was calculated as the mean item score divided by the maximum possible score for that item. P1 to P8 means passage 1 to passage 8. Scores on the argument strength evaluation were assigned on a 0–3 scale per flaw. Except for passage 1, which includes two flaws (full score = 6 points), all other passages have a full score of 3 points. For the argument relationship analysis, most passages have a full score of 2 points (1 point for correct relationship and 1 point for correct reason). Passage 2 has a full score of 3 points (1 point for identifying the refutation of passage 1, and 2 points for two reasons), and passage 8 has a full score of 4 points (2 points for identifying the refutations of passages 4 and 7, and 2 points for respective reasons).

**Table 3:** Four ability profiles’ performance on criterion measures.

Profile	TSA	EWCTET	Number Series Reasoning	Matrix Reasoning	Spatial Reasoning
1 Comprehensive advantage	0.01	0.04	0.02	0.02	0.04
2 Comprehensive disadvantage	−0.05	−0.07	−0.06	−0.08	−0.06
3 Balanced but relatively weak in relationship analysis	0.01	0.00	0.00	0.00	−0.01
4 Balanced but relatively strong in relationship analysis	−0.01	−0.02	0.01	0.02	0.01

Note: Ability estimates on each measure were standardized with a mean of zero; values above zero therefore indicate that a profile’s mean exceeds the overall sample mean, and values below zero indicate the converse.

**Table 4:** Correlations of the ability dimensions in the IAT with criterion tests.

	Single-Round Argument Analysis Ability	Inter-Argument Relationship Analysis Ability	Argument Integration Ability	TSA	EWCTET	Number Series Reasoning	Matrix Reasoning
Inter-argument relationship analysis ability	0.43 ***						
Argument integration ability	0.31 ***	0.06					
TSA	0.28 ***	0.23 ***	0.11 *				
EWCTET	0.33 ***	0.22 ***	0.12 *	0.33 ***			
Number series reasoning	0.21 ***	0.21 ***	0.05	0.58 ***	0.25 ***		
Matrix reasoning	0.20 ***	0.19 ***	0.01	0.53 ***	0.27 ***	0.87 ***	
Spatial reasoning	0.11 *	0.09	−0.04	0.20 ***	0.22 ***	0.39 ***	0.62 ***

Note: *** *p* < .001, * *p* < .05.

**Table 5:** Comparison of ability between debater sample and general sample.

	Debater Sample			General Sample		
	Mean	Min	Max	Mean	Min	Max
Single-round argument analysis	0.55	−0.65	0.99	0.00	−1.26	0.81
Inter-argument relationship analysis	0.41	−0.34	1.03	0.00	−0.57	0.54
Argument integration	0.17	−0.50	0.59	0.00	−0.68	0.62
EWCTET	0.51	0.30	0.63	0.00	−0.47	0.24
TSA	−0.02	−0.22	0.08	0.00	−0.46	0.08

**Table 6:** Proportion of four ability profiles among debaters and general sample.

Profile	Debater	General
1 Comprehensive advantage	76%	26%
2 Comprehensive disadvantage	3%	11%
3 Balanced but relatively weak in relationship analysis	18%	41%
4 Balanced but relatively strong in relationship analysis	3%	21%

**Figure A1:**
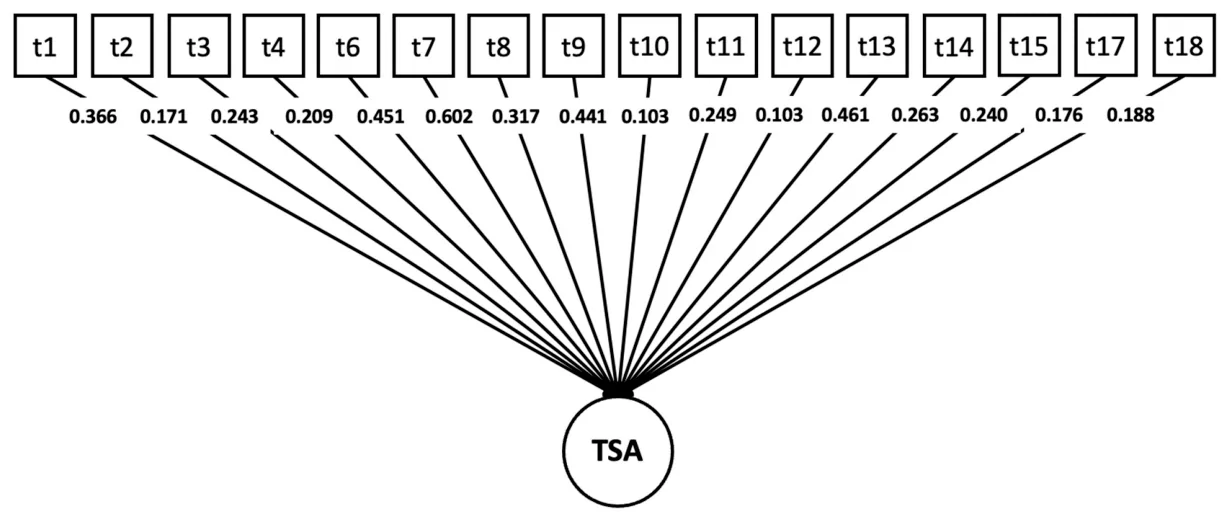
CFA results of the TSA.

**Figure A2:**
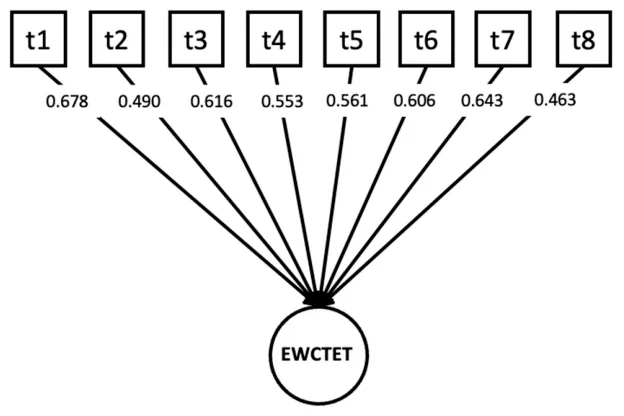
CFA results of the EWCTET.

**Figure A3:**
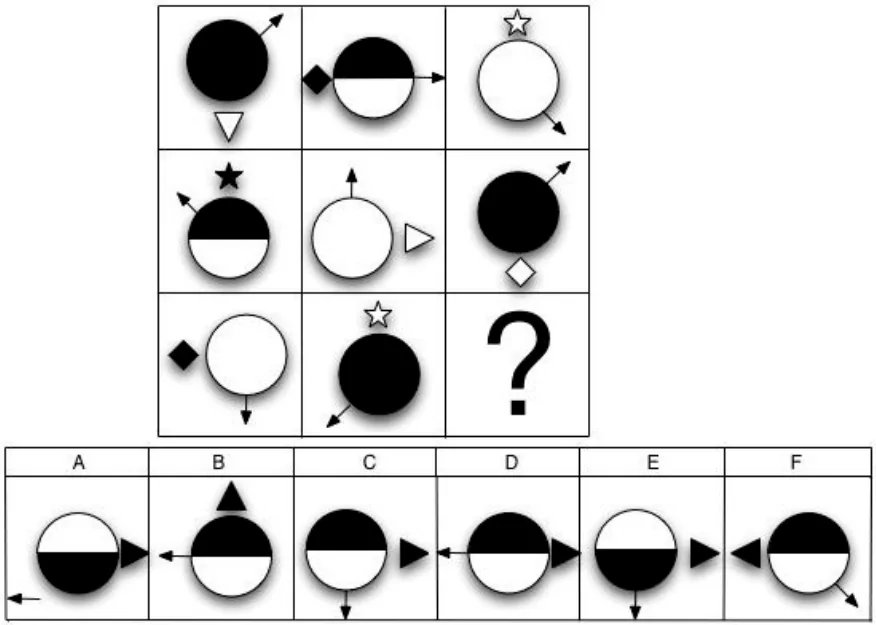
Example item of number series reasoning.

**Figure A4:**
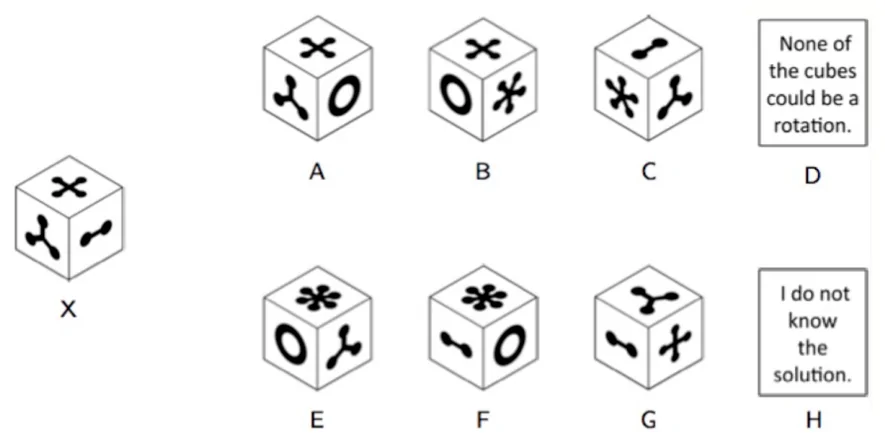
Example item of spatial reasoning.

**Figure A5:**
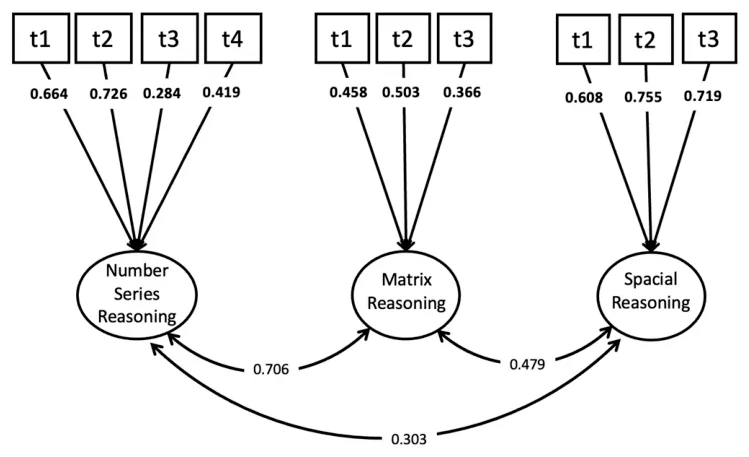
CFA results of the reasoning test.

## Data Availability

All data and code used in this study are openly available at: https://github.com/Jiang-Liming/Materials-for-IAT-paper (accessed on 16 August 2026).

## Note

1 The IAT is implemented through web programming, which can theoretically run in any computer environment with a browser and Internet. Accessing it requires obtaining a test account, so any researcher who wants to use it can contact the corresponding author. It should be noted that IAT currently only supports the Chinese version. We welcome scholars from various language backgrounds to jointly develop versions suitable for their language and culture.

## Abbreviations

The following abbreviations are used in this manuscript:

IAT	Iterative Argumentation Task
TSA	Thinking Skill Assessment
EWCTET	Ennis–Weir Critical Thinking Essay Test
MBTI	Myers–Briggs Type Indicator
LLM	large language model
ICC	intraclass correlation coefficients
CFA	confirmatory factor analysis
LPA	latent profile analysis

## Appendix A. Item Examples and CFA Results of Criterion Tests

TSA item example:

Rhona is a self-employed IT technician. Some of her clients are also friends. When she checks a computer for problems, she sees what is stored on the hard disk. She has sometimes teased friends in front of others about the websites they have been visiting and the pictures she has seen stored on their computers. She should not have done this, because it is wrong for people to reveal sensitive information which they know only because of their work. Which one of the following illustrates the principle used in the above argument?

A. Anyone who, in the course of their work, discovers evidence of terrorism should report it to the police.

B. Police officers should be dismissed if they pass confidential information to journalists about cases they have investigated.

C. Doctors and nurses should have access to patients’ medical records in order to give them the correct advice and treatment.

D. Ex-boyfriends and ex-girlfriends should not share personal or intimate information about their former partners, because such knowledge is private.

E. Teachers often come to know quite a lot of information about the families of their pupils, which they should report if they have any concerns.

A single-factor CFA was conducted on the 18 items of the TSA (Figure A1). Two items with factor loading close to zero were excluded. The fit indices for the final model were as follows: *χ*^2^ = 133.661, *df* = 104, *p* = .027, CFI = 0.886, TLI = 0.868, RMSEA = 0.028, and SRMR = 0.049.

EWCTET item example:

Last month, the Chief of Police, Burgess Jones, ran an experiment which proves that parking should be prohibited from 2 a.m. to 6 a.m. On one of our busiest streets, Marquand Avenue, he placed experimental signs for one day. The signs prohibited parking from 2 a.m. to 6 a.m. During the four-hour period, there was not one accident on Marquand. Everyone knows, of course, that there have been over four hundred accidents in Marquand during the past year.

A single-factor CFA was conducted on the eight items (eight paragraphs) of the EWCTET (Figure A2) and no item was removed. The fit indices for the model were as follows: *χ*^2^ = 52.816, *df* = 20, *p* < .001, CFI = 0.952, TLI = 0.932, RMSEA = 0.065, and SRMR = 0.042.

Number series reasoning item example:

What follows in the sequence?

8, 13, 21, 34, 55 __

Matrix reasoning item example:

As illustrated in Figure A3, participants are required to deduce the underlying pattern within the large matrix and select the correct option to complete the missing part.

Spatial reasoning item example:

As illustrated in Figure A4, each face of Cube X features a distinct pattern, and participants are required to select the option that represents a valid rotation of the cube.

A three-factor CFA was conducted on the reasoning test (Figure A5) and no item was removed. The fit indices for the model were as follows: *χ*^2^ = 43.276, *df* = 32, *p* = .088, CFI = 0.978, TLI = 0.969, RMSEA = 0.032, and SRMR = 0.038.

## Appendix B. Evidence Identification by LLM

In this study, an LLM (ERNIE-4) was employed to code evidence within in-process argumentative texts. The LLM coding procedure consisted of two steps, and the accuracy of each step was validated.

Step 1: Identification of newly added content.

First, pre- and post-revision argumentative texts of the participants were concatenated, preceded by the following prompt: “Identify the content added in the new text compared to the old text. Output only the added content. If there is no added content, output ‘None’.” A manual verification was conducted on 200 randomly sampled instances of newly added content extracted by the LLM. The results revealed an accuracy of 98.5%, indicating a high level of precision in recognizing new content.

Example:

Prompt:

Identify the content added in the new text compared to the old text. Output only the added content. If there is no added content, output ‘None’.

Old text: I believe that MBTI can, to some extent, help people better understand themselves. First, it is a personality test based on psychological indicators, which can help people understand their tendencies across four dimensions and thus choose learning and life styles, rhythms, and approaches that better suit them.

New text: I believe that MBTI can, to some extent, help people better understand themselves. First, it is a personality test based on psychological indicators, which can help people understand their tendencies across four dimensions and thus choose learning and life styles, rhythms, and approaches that better suit them. Although its developers were not professional psychologists, their backgrounds in literature and education also provided some support for the development of this test.

Output:

Although its developers were not professional psychologists, their backgrounds in literature and education also provided some support for the development of this test.

The instruction for manual coding:

Please compare the old text and the new text and determine whether the newly added text is accurate. An extraction should be coded as accurate if it comprehensively includes all substantive newly added content without omitting meaningful additions. Label 1 if the extraction is accurate; otherwise, label 0.

Step 2: Evidence coding of the newly added content.

First, the newly added content was concatenated with one of 11 pieces of evidence, preceded by the prompt: “Below is a piece of evidence regarding MBTI and a text segment. Please determine whether the text directly cites the content of the evidence. Output only ‘Yes’ or ‘No’.” This input was fed into the LLM to generate responses. To evaluate the accuracy, 100 instances of “Yes” outputs and 100 instances of “No” outputs from the LLM were randomly sampled for manual verification. The accuracy of “No” outputs ranged from 98% to 100% across 11 evidence categories. This demonstrates that the LLM is highly accurate in determining when a text does not contain specific evidence; therefore, the “No” codings by the LLM were all adopted. Conversely, the accuracy of “Yes” outputs was relatively low for certain evidence categories. After examination, the lower accuracy of “Yes” outputs was primarily driven by false positives (i.e., misclassifying text into a category it does not belong to). A manual review was conducted on all instances coded as “Yes” by the LLM. These verified results were subsequently utilized as the final coding.

Example 1: Newly added text contains the corresponding evidence

Prompt:

Below is a piece of evidence regarding MBTI and a text segment. Please determine whether the text directly cites the content of the evidence. Output only ‘Yes’ or ‘No’.

Evidence: The developers of MBTI were Katharine Cook Briggs and her daughter Isabel Briggs Myers, neither of whom was formally trained as a psychologist. Katharine was an educator, and Isabel was a writer. After developing an interest in psychology, they began studying Jung’s work and developed the MBTI. These details are documented in The Personality Brokers. In other words, MBTI was developed by psychology enthusiasts rather than professional psychologists.

Text: Although its developers were not professional psychologists, their backgrounds in literature and education also provided some support for the development of this test.

Output:

Yes

Example 2: Newly added text does not contain the corresponding evidence

Prompt:

Below is a piece of evidence regarding MBTI and a text segment. Please determine whether the text directly cites the content of the evidence. Output only ‘Yes’ or ‘No’.

Evidence: Many large companies, such as McKinsey, also use MBTI. If MBTI were useless, these large companies would certainly not use it.

Text: Although its developers were not professional psychologists, their backgrounds in literature and education also provided some support for the development of this test.

Output:

No

The instruction for manual coding:

Please determine whether the newly added text contains the corresponding evidence. Code the instance as 1 if the newly added text directly quotes, paraphrases, or summarizes the core content of the evidence. Code the instance as 0 if the newly added text does not contain the content of the evidence.

## Appendix C. Scoring Rubric in Argument Writing

**Table A1:** Scoring rubric in argument writing.

Score	Description
Clarity of claim: Whether the writing presents a clear and explicit position. If the claim is unclear or ambiguous, a low score should be given.	
Score = 1	No claim is presented.
Score = 2	No explicit claim is presented, but the author’s intended position can be inferred from the writing.
Score = 3	A claim is present but is not sufficiently clear or consistent. Some content deviates from the claim, or the position shifts through the writing.
Score = 4	A claim is present and generally clear but may lack perfect consistency. A small portion of the content may deviate from the claim.
Score = 5	A clear, explicit, and consistent claim is presented. The entire argument consistently supports this claim.
Sufficiency of evidence: Whether the writing provides adequate evidence to support the claim. If the claim lacks supporting evidence, a low score should be given.	
Score = 1	No evidence is provided, or the evidence used is clearly unreasonable or invalid.
Score = 2	Limited evidence is provided (e.g., 1–2 pieces), and the quality is insufficient. Some sub-arguments may lack evidence.
Score = 3	2–3 pieces of evidence are provided, with acceptable quality. Some sub-arguments may lack evidence.
Score = 4	2–3 pieces of evidence are provided, with generally high quality. Each sub-argument is supported.
Score = 5	3 or more pieces of evidence are provided, all of high quality. Each sub-argument is supported.
Adequacy of elaboration: Whether the writing sufficiently explains how the evidence supports the claim. If the writing merely lists evidence without elaboration, a low score should be given.	
Score = 1	Evidence is simply listed without any elaboration.
Score = 2	Evidence is listed with minimal elaboration (e.g., the main body consists of listed evidence, with overall elaboration at the beginning or end of the writing).
Score = 3	Some pieces of evidence are elaborated but not every piece, and overall elaboration is also present.
Score = 4	Most pieces of evidence have corresponding elaboration, and overall elaboration is also present.
Score = 5	Nearly all pieces of evidence have corresponding elaboration, and overall elaboration is also present.
Coherence of structure: Whether the writing demonstrates a clear and logical structure, such as well-defined relationships between paragraphs and sub-arguments, and smooth logical flow throughout the writing. If paragraphs are disorganized or logic is unclear, a low score should be given.	
Score = 1	Evidence is simply listed without paragraph structure.
Score = 2	The writing is organized in paragraph form, but often consists of only one paragraph, and the content within is disorganized.
Score = 3	Paragraphs are present, but the function of each paragraph is not clearly defined, and the content within is somewhat disorganized.
Score = 4	Paragraphs are relatively clear, and the function of each paragraph is fairly well defined, but the sub-points within each paragraph lack clarity.
Score = 5	Paragraphs are clear, the function of each paragraph is well defined, and each paragraph contains clearly organized sub-points.
Fairness: Whether the writing includes discussion of both sides of the argument, and whether the discussion of the opposing view is adequate and fair. If the essay only discusses one’s own position, or if the discussion of the opposing view is inadequate or biased, a low score should be given.	
Score = 1	The opposing view is not addressed at all.
Score = 2	The opposing view is briefly mentioned without substantive discussion.
Score = 3	The opposing view is discussed, but only 1–2 aspects are addressed, and the discussion may be inadequate or contain some bias.
Score = 4	The opposing view is discussed, covering 2–3 aspects. The discussion is relatively thorough with minimal bias.
Score = 5	The opposing view is discussed, covering 3 or more aspects. The discussion is thorough and unbiased.
